# Supplementary material for: Smooth Interpolating Curves with Local Control and Monotone Alternating Curvature
Source: Comput Graph Forum. 2022 Oct 6;41(5):25–38. doi: 10.1111/cgf.14600 (PMC9827861; doi:10.1111/cgf.14600)
Supplement: Supplementary file 1 — Supplement Material [file CGF-41-25-s001.zip › Local-Smooth-Interpolating-MonoCurvature/extern/clothoids/docs/api-matlab/program_listing_file_Biarc.m.html]

Program Listing for File Biarc.m — Clothoids v2.0.9

### Navigation

- index
- toc
- Clothoids »
- Program Listing for File Biarc.m

# Program Listing for File Biarc.m¶

↰ Return to documentation for file (`Biarc.m`)

```
classdef Biarc < CurveBase
  %> MATLAB class wrapper for the underlying C++ class

  methods
    %> Create a new C++ class instance for the Biarc object
    %>
    %> **Usage:**
    %>
    %> \rst
    %> .. code-block:: matlab
    %>
    %>   self = Biarc();
    %>   self = Biarc( x0, y0, theta0, x1, y1, theta1 );
    %>
    %> \endrst
    %>
    %> **Optinal Arguments:**
    %>
    %> - `x0`, `y0`: coordinate of initial point
    %> - `theta0`    : orientation of the clothoid at initial point
    %> - `x1`, `y1`: coordinate of final point
    %> - `theta1`    : orientation of the clothoid at final point
    %>
    %> **On output:**
    %>
    %> - self: reference handle to the object instance
    %>
    function self = Biarc( varargin )
      self@CurveBase( 'BiarcMexWrapper' );
      self.objectHandle = BiarcMexWrapper( 'new' );
      if nargin > 0
        ok = BiarcMexWrapper( 'build_G1', self.objectHandle, varargin{:} );
        if ~ok
          error('Biarc constructor failed');
        end
      end
    end
    % - - - - - - - - - - - - - - - - - - - - - - - - - - - - - - - - - - -
    %> Build the interpolating G1 biarc
    %>
    %> **Usage:**
    %>
    %> \rst
    %> .. code-block:: matlab
    %>
    %>   ref.build_G1( x0, y0, theta0, x1, y1, theta1 );
    %>
    %> \endrst
    %>
    %> **On input:**
    %>
    %> - `x0`, `y0`: coordinate of initial point
    %> - `theta0`    : orientation of the clothoid at initial point
    %> - `x1`, `y1`: coordinate of final point
    %> - `theta1`    : orientation of the clothoid at final point
    %>
    function ok = build( self, x0, y0, theta0, x1, y1, theta1 )
      ok = BiarcMexWrapper( 'build_G1', self.objectHandle, ...
                            x0, y0, theta0, x1, y1, theta1 );
    end
    % - - - - - - - - - - - - - - - - - - - - - - - - - - - - - - - - - - -
    function str = is_type( ~ )
      str = 'BiArc';
    end
    % - - - - - - - - - - - - - - - - - - - - - - - - - - - - - - - - - - -
    %> Build the interpolating biarc by 3 points
    %>
    %> **Usage:**
    %>
    %> \rst
    %> .. code-block:: matlab
    %>
    %>   ref.build_3P( x0, y0, x1, y1, x2, y2 );
    %>   ref.build_3P( [x0, y0], [x1, y1], [x2, y2] );
    %>
    %> \endrst
    %>
    %> **On input:**
    %>
    %> - `x0`, `y0`: coordinate of initial point
    %> - `x1`, `y1`: coordinate of middle point
    %> - `x2`, `y2`: coordinate of final point
    %>
    %> alternative
    %>
    %>    p0: coordinate of initial point
    %>    p1: coordinate of middle point
    %>    p2: coordinate of final point
    %>
    function ok = build_3P( self, varargin )
      ok = BiarcMexWrapper( 'build_3P', self.objectHandle, varargin{:} );
    end
    % - - - - - - - - - - - - - - - - - - - - - - - - - - - - - - - - - - -
    %> Get junction point of the biarc x-coordinate
    %>
    %> **Usage:**
    %>
    %> \rst
    %> .. code-block:: matlab
    %>
    %>   x = ref.xMiddle();
    %>
    %> \endrst
    %>
    function res = xMiddle( self )
      res = BiarcMexWrapper( 'xMiddle', self.objectHandle );
    end
    % - - - - - - - - - - - - - - - - - - - - - - - - - - - - - - - - - - -
    %> Get junction point of the biarc y-coordinate
    %>
    %> **Usage:**
    %>
    %> \rst
    %> .. code-block:: matlab
    %>
    %>   x = ref.yMiddle();
    %>
    %> \endrst
    %>
    function res = yMiddle( self )
      res = BiarcMexWrapper( 'yMiddle', self.objectHandle );
    end
    % - - - - - - - - - - - - - - - - - - - - - - - - - - - - - - - - - - -
    %> Get junction point angle of the biarc
    %>
    %> **Usage:**
    %>
    %> \rst
    %> .. code-block:: matlab
    %>
    %>   theta = ref.thetaMiddle();
    %>
    %> \endrst
    %>
    function res = thetaMiddle( self )
      res = BiarcMexWrapper( 'thetaMiddle', self.objectHandle );
    end
    % - - - - - - - - - - - - - - - - - - - - - - - - - - - - - - - - - - -
    %> Get curvature of the first arc of the biarc
    %>
    %> **Usage:**
    %>
    %> \rst
    %> .. code-block:: matlab
    %>
    %>   kappa0 = ref.kappa0();
    %>
    %> \endrst
    %>
    function res = kappa0( self )
      res = BiarcMexWrapper( 'kappa0', self.objectHandle );
    end
    % - - - - - - - - - - - - - - - - - - - - - - - - - - - - - - - - - - -
    %> Get curvature of the second arc of the biarc
    %>
    %> **Usage:**
    %>
    %> \rst
    %> .. code-block:: matlab
    %>
    %>   kappa1 = ref.kappa1();
    %>
    %> \endrst
    %>
    function res = kappa1( self )
      res = BiarcMexWrapper( 'kappa1', self.objectHandle );
    end
    % - - - - - - - - - - - - - - - - - - - - - - - - - - - - - - - - - - -
    %> Get length of the first arc of the biarc
    %>
    %> **Usage:**
    %>
    %> \rst
    %> .. code-block:: matlab
    %>
    %>   length0 = ref.length0();
    %>
    %> \endrst
    %>
    function res = length0( self )
      res = BiarcMexWrapper( 'length0', self.objectHandle );
    end
    % - - - - - - - - - - - - - - - - - - - - - - - - - - - - - - - - - - -
    %> Get length of the second arc of the biarc
    %>
    %> **Usage:**
    %>
    %> \rst
    %> .. code-block:: matlab
    %>
    %>   length1 = ref.length1();
    %>
    %> \endrst
    %>
    function res = length1( self )
      res = BiarcMexWrapper( 'length1', self.objectHandle );
    end
    % - - - - - - - - - - - - - - - - - - - - - - - - - - - - - - - - - - -
    %> Get the biarc G1 data
    %>
    %> **Usage:**
    %>
    %> \rst
    %> .. code-block:: matlab
    %>
    %>   [x0,y0,theta0,x1,y1,theta1] = ref.getData();
    %>
    %> \endrst
    %>
    %> - `x0`, `y0`: initial point of the biarc
    %> - `theta0`    : initial angle of the biarc
    %> - `x1`, `y1`: final point of the biarc
    %> - `theta1`    : final angle of the biarc
    %>
    function [ x0,y0,theta0,x1,y1,theta1] = getData( self )
      x0     = self.xBegin();
      y0     = self.yBegin();
      theta0 = self.thetaBegin();
      x1     = self.xEnd();
      y1     = self.yEnd();
      theta1 = self.thetaEnd();
    end
    % - - - - - - - - - - - - - - - - - - - - - - - - - - - - - - - - - - -
    %> Get the two circle arc composing a biarc
    %>
    %> **Usage:**
    %>
    %> \rst
    %> .. code-block:: matlab
    %>
    %>   [C0,C1] = ref.getCircles();
    %>
    %> \endrst
    %>
    function [ C0, C1 ] = getCircles( self )
      x0     = self.xBegin();
      y0     = self.yBegin();
      theta0 = self.thetaBegin();
      kappa0 = self.kappa0();
      L      = self.length0();
      C0     = CircleArc( x0, y0, theta0, kappa0, L );
      x0     = self.xMiddle();
      y0     = self.yMiddle();
      theta0 = self.thetaMiddle();
      kappa0 = self.kappa1();
      L      = self.length1();
      C1     = CircleArc( x0, y0, theta0, kappa0, L );
    end
    % - - - - - - - - - - - - - - - - - - - - - - - - - - - - - - - - - - -
    %> Return the nurbs represantation of the two arc composing the biarc
    %>
    %> **Usage:**
    %>
    %> \rst
    %> .. code-block:: matlab
    %>
    %>   [arc0,arc1] = ref.to_nurbs();
    %>
    %> \endrst
    %>
    %> - `arc0`: the nurbs of the first arc
    %> - `arc1`: the nurbs of the second arc
    %>
    function [ arc0, arc1 ] = to_nurbs( self )
      [ arc0, arc1 ] = BiarcMexWrapper( 'to_nurbs', self.objectHandle );
    end
    % - - - - - - - - - - - - - - - - - - - - - - - - - - - - - - - - - - -
    %> Get the curvilinear coordinates of the point `(x,y)`
    %>
    %> **Usage:**
    %>
    %> \rst
    %> .. code-block:: matlab
    %>
    %>   [s,t] = ref.find_coord( x, y );
    %>
    %> \endrst
    %>
    %> - `s`: curvilinear coordinate along the curve
    %> - `t`: curvilinear coordinate along the normal of the curve
    %>
    function [ s, t ] = find_coord( self, x, y )
      [ s, t ] = BiarcMexWrapper( 'findST', self.objectHandle, x, y );
    end
    % - - - - - - - - - - - - - - - - - - - - - - - - - - - - - - - - - - -
    %> Plot the biarc
    %>
    %> **Usage:**
    %>
    %> \rst
    %> .. code-block:: matlab
    %>
    %>   ref.plot( npts );
    %>
    %>   fmt1 = {'Color','blue','Linewidth',2};
    %>   fmt2 = {'Color','red','Linewidth',2};
    %>   ref.plot( npts, fmt1, fmt2 );
    %>
    %> \endrst
    %>
    %> - `npts`: number of sampling points for plotting
    %> - `fmt1`: format of the first arc
    %> - `fmt2`: format of the second arc
    %>
    function plot( self, npts, varargin )
      if nargin<2
        npts = 64;
      end
      if nargin>2
        fmt1 = varargin{1};
      else
        fmt1 = {'Color','blue','Linewidth',2};
      end
      if nargin>3
        fmt2 = varargin{2};
      else
        fmt2 = {'Color','red','Linewidth',2};
      end
      [C0,C1] = self.getCircles();
      C0.plot(npts,fmt1);
      hold on;
      C1.plot(npts,fmt2);
    end
    % - - - - - - - - - - - - - - - - - - - - - - - - - - - - - - - - - - -
    %> Plot the curvature of the biarc
    %>
    %> **Usage:**
    %>
    %> \rst
    %> .. code-block:: matlab
    %>
    %>   ref.plotCurvature( npts );
    %>
    %>   fmt1 = {'Color','blue','Linewidth',2};
    %>   fmt2 = {'Color','red','Linewidth',2};
    %>   ref.plotCurvature( npts, fmt1, fmt2 );
    %>
    %> \endrst
    %>
    %> - `npts`: number of sampling points for plotting
    %> - `fmt1`: format of the first arc
    %> - `fmt2`: format of the second arc
    %>
    function plotCurvature( self, npts, varargin )
      if nargin < 2
        npts = 1000;
      end
      L = BiarcMexWrapper( 'length', self.objectHandle );
      S = 0:L/npts:L;
      [ ~, ~, ~, kappa ] = BiarcMexWrapper( 'evaluate', self.objectHandle, S );
      plot( S, kappa, varargin{:} );
    end
    % - - - - - - - - - - - - - - - - - - - - - - - - - - - - - - - - - - -
    %> Plot the angle of the biarc
    %>
    %> **Usage:**
    %>
    %> \rst
    %> .. code-block:: matlab
    %>
    %>   ref.plotAngle( npts );
    %>
    %>   fmt1 = {'Color','blue','Linewidth',2};
    %>   fmt2 = {'Color','red','Linewidth',2};
    %>   ref.plotAngle( npts, fmt1, fmt2 );
    %>
    %> \endrst
    %>
    %> - `npts`: number of sampling points for plotting
    %> - `fmt1`: format of the first arc
    %> - `fmt2`: format of the second arc
    %>
    function plotAngle( self, npts, varargin )
      if nargin < 2
        npts = 1000;
      end
      L = BiarcMexWrapper( 'length', self.objectHandle );
      S = 0:L/npts:L;
      [ ~, ~, theta, ~ ] = BiarcMexWrapper( 'evaluate', self.objectHandle, S );
      plot( S, theta, varargin{:} );
    end
    % - - - - - - - - - - - - - - - - - - - - - - - - - - - - - - - - - - -
    %> Plot the normal of the biarc
    %>
    %> **Usage:**
    %>
    %> \rst
    %> .. code-block:: matlab
    %>
    %>   ref.plotNormal( step, len );
    %>
    %> \endrst
    %>
    %> - `step`: number of sampling normals
    %> - `len`:  length of the plotted normal
    %>
    function plotNormal( self, step, len )
      for s=0:step:self.length()
        [ x, y, theta, ~ ] = self.evaluate(s);
        n = [sin(theta),-cos(theta)];
        A = [x,x+len*n(1)];
        B = [y,y+len*n(2)];
        plot(A,B);
      end
    end
  end
end
```

### Quick search

### Table of Contents

- Matlab Interface Manual
- C++ API
- MATLAB API

«
hide menu

menu
sidebar
»

### Navigation

- index
- toc
- Clothoids »
- Program Listing for File Biarc.m

© Copyright 2021, Enrico Bertolazzi and Marco Frego.
Created using Sphinx 4.2.0.
